# Effects of dietary flavonoids on performance, blood constituents, carcass composition and small intestinal morphology of broilers: a meta-analysis

**DOI:** 10.5713/ajas.20.0379

**Published:** 2020-08-24

**Authors:** Tri Rachmanto Prihambodo, Muhammad Miftakhus Sholikin, Novia Qomariyah, Anuraga Jayanegara, Irmanida Batubara, Desianto Budi Utomo, Nahrowi Nahrowi

**Affiliations:** 1 Graduate Study Program of Nutrition and Feed Science, Faculty of Animal Science, IPB University, Bogor 16680, Indonesia; 2 Animal Feed and Nutrition Modelling (AFENUE) Research Group, Faculty of Animal Science, IPB University, Bogor 16680, Indonesia; 3 South Sulawesi Assessment Institute of Agriculture Technology, Makassar 90242, Indonesia; 4 Department of Nutrition and Feed Technology, Faculty of Animal Science, IPB University, Bogor 16680, Indonesia; 5 Department of Chemistry, Faculty of Mathematics and Natural Sciences, IPB University, Bogor 16680, Indonesia; 6 PT. Charoen Pokphand, Jakarta 14430, Indonesia

**Keywords:** Broiler, Flavonoid, Growth Performance, Carcass and Blood Composition, Small Intestinal Morphology, Meta-analysis

## Abstract

**Objective:**

This study aims to evaluate the influence of dietary flavonoids on the growth performance, blood and intestinal profiles, and carcass characteristics of broilers by employing a meta-analysis method.

**Methods:**

A database was built from published studies which have reported on the addition of various levels of flavonoids from herbs into broiler diets and then monitored growth performance, blood constituents, carcass proportion and small intestinal morphology. A total of 42 articles were integrated into the database. Several forms of flavonoids in herbs were applied in the form of unextracted and crude extracts. The database compiled was statistically analyzed using mixed model methodology. Different studies were considered as random effects, and the doses of flavonoids were treated as fixed effects. The model statistics used were the p-values and the Akaike information criterion. The significance of an effect was stated when its p-value was <0.05.

**Results:**

Dietary flavonoids increased (quadratic pattern; p<0.05) the average daily gain of broilers in the finisher phase. There was a reduction (p<0.01) in the feed conversion ratio of the broilers both in the starter (linear pattern) and finisher phases (quadratic pattern). The mortality rate tended to decrease linearly (p<0.1) with the addition of flavonoids, while the carcass parameter was generally not influenced. A reduction (p<0.001) in cholesterol and malondialdehyde concentrations (both linearly) was observed, while super oxide dismutase activity increased linearly (p<0.001). Increasing the dose of flavonoids increased (p<0.01) the villus height (VH) and villus height and crypt depth (VH:CD) ratio (p<0.05) in the duodenum. Similarly, the VH:CD ratio was elevated (p<0.001) in the jejunum following flavonoid supplementation.

**Conclusion:**

Increasing levels of flavonoids in broilers diet leads to an improvement in growth performance, blood constituents, carcass composition and small intestinal morphology.

## INTRODUCTION

Two main goals of the broiler industry are to improve growth performance and optimize the quality of broiler meat. In addition, another objective at present is to produce healthy broiler meat without the addition of any antibiotic growth promoters. This is considered to be important, as regulations in many countries prohibit the use of antibiotics and hormones for growth [1]. However, the lack of antibiotic use in broiler production, especially in tropical countries, may potentially interfere with performance and tend to increase death rates among broilers because of their diminished health status [2]. Broiler production in the tropics faces greater obstacles than in sub-tropical or temperate regions, such as lower growth performance, depressed immune systems, low carcass quality and high mortality rates due to the higher temperatures and humidity, which lead to an enhanced heat stress response [3–5]. Some of the alternatives to antibiotics to overcome such problems are plant secondary metabolites such as tannins, flavonoids, saponins and essential oils [6]. These natural plant compounds have been reported to be able to replace antibiotics without negatively interfering with the main production parameters of broiler [6–8].

One of the potential secondary metabolites for use in broiler production are flavonoids, a compound that can be found in various parts of plants, such as the fruits, leaves, stems, and bark. It had been reported that flavonoids can be used as human and animal medicine since the compound possesses antibacterial, antioxidant, antiinflammation and hepatoprotective properties [9,10]. Flavonoids have positive effects on the digestive tract and cardiovascular system, stimulate the release of insulin hormone [11], modulate lipid metabolism, and improve antioxidant activity in the carcass of broiler. However, they have also been reported to have some negative effects, such as the possibility of acting as mutagens, pro-oxidants that generate free radicals, and as inhibitors of key enzymes involved in hormone metabolism when fed in high dosages [12]. Despite a number of experiments regarding the influence of flavonoids on broilers, to date there has been no meta-analysis study which has attempted to quantitatively summarize such a relationship. Therefore, the aim of this paper was to evaluate the influence of dietary flavonoids on growth performance, blood and intestinal profiles, and carcass characteristics of broilers by employing a meta-analysis method.

## MATERIALS AND METHODS

### Development of the database

A database was built from published studies which have reported on the addition of various levels of flavonoids from herbs to broiler diets. Accordingly, different forms and levels of flavonoid supplementation, as well as various flavonoid sources, were specified in the database. Science Direct, Google Scholar and Scopus were used as the search tools to compile the related articles, using the keywords “flavonoid”, “herbs”, and “broiler”. Growth performance (body weight, average daily gain [ADG], daily feed intake, feed conversion ratio [FCR], and mortality); blood constituents (glucose, cholesterol, triglyceride, high-density lipoprotein [HDL], low-density lipoprotein [LDL], malondialdehyde [MDA], and superoxide dismutase); carcass proportion (weight, breast, leg, and abdominal fat); and small intestinal morphology (villus height [VH], crypt depth [CD], and ratio of villus height to crypt depth [VH:CD]) were the parameters included in the database.

The criteria for articles to be included in the database were that: i) they were published in English; ii) treatments included the addition of a flavonoid source to certain basal feeds; and iii) experiments were conducted *in vivo* on broilers. A total of 60 articles were initially found after searching using the above-mentioned keywords. All these papers were further evaluated on the basis of their abstracts and full texts, which resulted in 42 papers being integrated into the database (Table 1). When an article reported more than one experiment, each of these was encoded separately. As indicated in Table 1, several forms of flavonoids in herbs were applied as unextracted and crude extracts. The supplementary doses ranged from 0 (control) to 60,000 mg/kg of diet. Broiler types integrated in the present study were Ross 308, Cobb 500 and Arbor Acres which are considered as fast-growing broilers. In the process of tabulating the data into the database, data for similar parameters were converted into the same measurement units, which facilitated further data analysis.

### Analysis of data

The database was statistically analyzed using mixed model methodology in which the model has been widely used in meta-analysis research related to animal nutrition [13,14]. Different studies were considered as random effects, and the doses of flavonoids were treated as fixed effects. The following statistical model was used:

$$Y_{ij}=B_{0}+B_{1}X_{ij}+B_{2}{X_{ij}}^{2}+s_{i}+b_{i}X_{ij}+e_{ij}$$

where *Y_ij_* = dependent variable, *B*_0_ = overall intercept across all studies (fixed effect), *B*_1_ = linear regression coefficient of Y on X (fixed effect), *B*_2_ = quadratic regression coefficient of Y on X (fixed effect), *X_ij_* = value of the continuous predictor variable (flavonoid addition level), *s_i_* = random effect of study *i*, *b_i_* = random effect of study *i* on the regression coefficient of *Y* on *X* in study *i*, *e_ij_* = the unexplained residual error. When the respective quadratic regression model was not significant at p<0.05, the corresponding linear regression mixed model was applied. Variable study was declared in the class statement since it does not contain any quantitative information. The model statistics used were p-values and the Akaike information criterion. The significance of an effect was stated when the p-value was <0.05. All statistical analyses were carried out using R software, version 3.60.

## RESULTS

The addition of flavonoids quadratically increased (p<0.05) the ADG of broilers in the finisher phase, but the effect was insignificant in the starter phase (Table 2). Feed intake was not affected at all by the addition of flavonoids. The addition reduced (p<0.01) the FCR of broilers both in the starter and finisher phases, in which the response was linear and quadratic, respectively. Based on the FCR parameter, the optimum flavonoid dosage for broilers was 0.83%. The mortality rate tended to decrease linearly (p<0.1) with the addition of flavonoids. Carcass-related parameters were generally not influenced by flavonoids (Table 3), apart from the fact that broiler liver weight increased (p<0.05).

The effects of flavonoids on blood constituents and the small intestinal morphology of broilers are presented in Table 4 and 5, respectively. Cholesterol and MDA concentrations decreased linearly (both at p<0.001), while super oxide dismutase activity increased (p<0.001) linearly due to higher doses of dietary flavonoids. The addition decreased (p<0.05) triglyceride and low-density lipoprotein (p<0.001) concentrations in the blood of the broilers, following quadratic patterns. On the contrary, high-density lipoprotein concentration was elevated (p<0.01) quadratically following the flavonoid addition. With regard to small intestinal morphology, higher flavonoid doses increased (p<0.01) VH and the VH:CD ratio (p<0.05) in the duodenum. Similarly, the VH:CD ratio was elevated (p<0.001) in the jejunum and ileum (p<0.1) due to the flavonoid supplementation.

## DISCUSSION

This meta-analysis study generally indicates the positive effects of dietary flavonoid addition on the growth performance, blood metabolites and small intestinal morphology of broilers. Flavonoids are a class of phytochemicals that possess antibacterial activity against a wide range of microbial species, including pathogenic and other undesirable bacteria present in the digestive tract. Pathogenic bacteria, as a main cause of disorders in the digestive tract, can apparently be inhibited by flavonoids. Such pathogens may injure the intestinal villi and therefore interfere with nutrient absorption [15]. Their population is reduced in the presence of flavonoids, leading to an improvement in the performance of broilers and a more efficient FCR. A lower pathogen community stimulates the growth and regeneration of intestinal villi and intensifies nutrient absorption. This is supported by the increase in the VH:CD ratio in the duodenum, jejunum, and ileum. The ratio itself is a histological index for intestinal digestive capacity; a higher value indicates that the chicken has better intestinal health and higher absorption capacity [16]. Therefore, promotion of performance results due to addition of flavonoids is inseparable from the high ratio of villi length and CD. In addition, they may stimulate mucus secretion [17] resulting in better villus protection and an increase in the growth of probiotic bacteria in the intestine [18].

The meta-analysis shows limited effects of flavonoids on the carcass and organ composition of broilers, except for the liver. This was confirmed by previous studies [19,20]. The increase in liver weight due to the addition of flavonoids is apparently a result of the detoxification process in the liver [21]. This implies that flavonoids should be added at an appropriate dosage so that they do not have any detrimental effect on broiler metabolism and production. Nevertheless, the liver weight in this study ranged from 1.71% to 3.88% of the total carcass weight, which is still within the normal weight range; the normal liver weight in broilers is between 2.64% to 4.40% of total carcass weight [22]. The effects of flavonoids on blood metabolites are in line with various previous studies, for example cholesterol reduction [23,24]; HDL enhancement [25,26]; LDL reduction [25,26]; MDA reduction [27] and superoxide dismutase enhancement [11,16]. Flavonoids are known to have the ability to modulate fat metabolism, changing the profile of fatty acids and the omega 3 to omega 6 ratio, thereby reducing cholesterol and triglyceride levels [11]. Zhang et al [28] reported that flavonoids reduced the content of CHO compounds and total glucose which are the constituents of LDL, apparently due to the delay in the activity of Acyl-CoA cholesterol acyltransferase in liver hepatocellular carcinoma cells [29]. Flavonoids possess antioxidant activity which is also able to modify lipid metabolism by inhibiting LDL formation [26], in which it is regarded as a bad cholesterol that may induce coronary heart disease.

With regard to blood glucose levels, flavonoids contain β-flavonoid rings [30] which play a role in glucose inhibition by forming hydrogen bonds with the active site of enzymes involved in carbohydrate digestion and metabolism. It has been reported that flavonoids inhibit the activity of α-glucosidase [31] and α-amylase [32], resulting in a slowdown of glucose absorption. The compound inhibits α-glucosidase, which converts disaccharide to monosaccharide, and inhibits α-amylase, which breaks down complex carbohydrates into monosaccharide. However, some studies have also reported that flavonoids had no effects on blood glucose level [33,34]. This meta-analysis indicates a non-significant effect of flavonoids on blood glucose, although the slope is clearly negative, most probably due to the low level of data available, thus leading to low statistical power.

## CONCLUSION

The meta-analysis has discovered that increasing flavonoid levels in broiler diets leads to an improvement in growth performance, blood constituents, carcass composition and small intestinal morphology, based on various literatures. Interestingly, the ADG increase due to dietary flavonoids is linked with improvement in small intestinal morphology, such as VH and the VH:CD ratio, without interfering with carcass composition. The concentration of blood constituents such as cholesterol, MDA, triglycerides, and low-density lipoprotein falls with higher doses of flavonoids, while increasing super oxide dismutase activity and high-density lipoprotein.

## Figures and Tables

**Table 1:** Studies included in the meta-analysis of flavonoid addition on performance, carcass, blood composition and small intestinal morphology of broilers

No	Reference	Period (d)	Source	Form	Flavonoid type	Dose (mg/kg)
1	Abolfathi et al [16]	1–42	Inula helenium	Crude extract	Kaempferol, quercetin	0–1,000
2	Akbarian et al [17]	22–38	Citrus aurantium	Crude extract	Quercetin, luteolin, rutin, and hesperitin	0–400
3	Daramola [18]	28–56	Vernonia amygdalina, Moringa oleifera	Unextracted	Quercetin	0–2,000
4	Kishawy et al [25]	1–42	Punica granatum	Crude extract	Myricetin, quercetin	0–1,000
5	Habibian et al [26]	1–49	Portulaca oleacea	Unextracted, crude extract	Kaempferol, quercetin	0–3,000
6	Ahmadipour and Khajali [27]	1–42	Urtica dioica	Unextracted	Quercetin, kaempferol, isorhamnetein	0–15,000
7	Abdel-Azeem and Basyony [35]	1–42	Alpinia galangal	Crude extract	Kaempferol, kaempferide, galangin, alpinin	0–750
8	Aengwanich et al [36]	28–35	Tamarindus indica	Crude extract	Quercetin	0–500
9	Arlette et al [37]	1–21	Ageratum conyzoides	Crude extract	Kaempferol, quercetin and glycosides	0–6,000
10	Bai et al [38]	1–41	Radix bupleuri, Radix astragali	Crude extract	Quercetin, isorhamnetin, isorhamnetin-3-O-glucoside, puerarin, rutin, narcissin, eugenin, saikochrome A, saikochromic acid, 7,4′-dihydroxy-isoflavone-7-O-β-D-glucoside, saikochromoside A and saikoisoflavonoside	0–800
11	Brenes et al [39]	1–42	Vitis vinifera	Crude extract	Catechin, epicatechin	0–360
12	Chamorro et al [40]	1–21	Vitis vinifera	Crude extract	Catechin, epicatechin	0–5,000
13	Dilawar et al [41]	1–35	Mentha arvensis, Geranium thunbergii	Crude extract	Quercetin	0–1,000
14	Dong et al [42]	1–28	Camelia oleifera	Unextracted	Kaempferol	0–500
15	Duarte et al [43]	1–42	Propolis	Crude extract	Quercetin, rutin, caffeic acid, chrysin, apigenin, and kaempferol	0–500
16	Goodarzi et al [44]	1–42	Allium cepa	Unextracted	Quercetin and isorhamnetein	0–30,000
17	Abdel-Moneim et al [45]	1–42	Punica granatum	Crude extract	Flavanols and anthocyanins	0–10
18	Herrero-Encinas et al [46]	22–42	Olea europaea	Crude extract	Hesperidin, rutin, luteolin-7-O-glucoside, apigenin, apigenin-7-O-glucoside, quercetin, kaempferol	0–750
19	Jobe et al [47]	1–96	Cassia abbreviate	Crude extract		0–500
20	Jiang et al [48]	1–42	Citri reticulatae pericarpium	Crude extract	Hesperidin	0–48
21	Khoobani et al [49]	1–42	Chicorium intybus	Unextracted	Kaempferol	0–2,000
22	Nardoia et al [50]	1–21	Vitis vinifera	Unextracted	Catechin and Epicatechin	0–60,000
23	Nkukwana et al [51]	1–42	Moringa oleifera	Unextracted	Kaempferol, quercetin, isorhamnetin, and apigenin	0–5,000
24	Oso et al [52]	1–42	Aerva lanata	Unextracted	Quercetin, kaempferol, and ferulic acid	0–5,000
25	Park et al [53]	1–35	Saposhnikovia divaricata	Crude extract	Rutin and ferulate	0–2,000
26	Park et al [53]	1–35	Scutellaria baicalensis	Crude extract	Quercetin and rutin	0–500
27	Park et al [54]	1–35	Saposhnikovia divaricate, Lonicera japonica, Chelidonium majus	Crude extract	Quercetin and rutin	0–1,000
28	Pirgozliev et al [55]	1–21	Carvacrol cinnamaldehyde	Unextracted	Quercetin	0–100
29	Placha et al [56]	1–28	Thymus vulgaris	Crude extract	Homoorientin, orientin, luteolin, kaempferol, luteolin, pratansein	0–1,000
30	Rahimi et al [57]	1–42	Thymus vulgaris, Echinacea purpurea, Allium sativum	Crude extract	Quercetin, kaempferol, delphinidin, myricetin, apigenin	0–1,000
31	Rashidi et al [58]	1–42	Glycyrrhiza glabra	Crude extract	Kaempferol, quercetin	0–500
32	Rubio et al [59]	1–21	Piper cubeba	Crude extract	Rutin, quercetin, luteolin, hesperidin, bioflavonoids	0–500
33	Saeid et al [60]	1–42	Zingiber officinale	Crude extract	Quercetin, rutin, catechin, epicatechin, kaempferol, and naringenin	0–6,000
34	Shen et al [61]	1–42	Bambusoideae	Crude extract	Isoflavone, quercetin	0–5,000
35	Shirzadi et al [62]	1–42	Prosopis farcta, Rhus coriaria	Crude extract	Vicenin-2, Apigenin C-glycoside, Iso-orientin (=Luteolin 6-C-glucoside), Myricetin 3-O-glucoside, Vitexin, Luteolin 7-O-glucoside, Isovitexin, Quercetin 3-O-glucoside, Rutin, Quercetin 3-O-galactoside, Chrysoeriol 7-O-glucoside, Kaempferol 3-O-rutonoside, Isorhamnetin 3-O-rutinoside, 5-deoxyluteolin, Caffeic acid derivative and Luteolin	0–200
36	Teteh et al [63]	1–28	Moringa oleifera	Crude extract	Kaempferol, quercetin, isorhamnetin, and apigenin	0–20,000
37	Vase-Khavari et al [64]	1–42	Rhus coriaria, H. persicum, M. piperita	Unextracted	Myricetin, quercetin, kaempferol, isoquercetin, rutin, afzelin, astragalin, isorhamnetin	0–5,000
38	Viveros et al [65]	1–21	Vitis vinifera	Crude extract	Catechin, epicatechin	0–7,200
39	Wang et al [66]	1–42	Forsythia suspensa	Crude extract	Quercetin, rutin	0–100
40	Zhang et al [67]	1–42	Ginkgo biloba	Crude extract	Quercetin, kaempferol, and isorhamnetin	0–500
41	Zhou et al [68]	1–42	Scutellaria baicalensis	Crude extract	Trihydroxyflavone	0–200

**Table 2:** Regression equations on the influence of flavonoid-rich herb addition (in % of diet as fed) on production performance of broilers

Response parameter	Model	n	Parameter estimates				Model estimates	
	
			Intercept	SE Intercept	Slope	SE Slope	p-value	AIC1)
Starter phase								
Bodyweight (g/bird)	L	107	1,055	108	35.1	20.1	0.085	1,352
ADG (g/bird/d)	L	101	41.7	2.32	1.30	0.83	0.120	601
Feed intake (g/bird/d)	L	105	63.4	3.51	−0.52	0.60	0.387	625
FCR (g/g)	L	99	1.54	0.06	−0.12	0.03	<0.001	−11.2
Mortality (%)	Q	16	11.4	2.47	−27.3	12.16	0.046	115
					193	45.4	<0.001	99.0
Finisher phase								
Bodyweight (g/bird)	Q	67	2,207	235	506	179	0.007	896
					−1,808	696	0.013	877
ADG (g/bird/d)	Q	78	80.6	7.19	9.95	5.77	0.090	556
					−64.9	26.8	0.019	544
Feed intake (g/bird/d)	L	78	152	11.4	10.0	6.31	0.118	607
FCR (g/g)	Q	78	1.87	0.05	−0.11	0.09	0.246	−99.0
					1.27	0.43	0.005	105
Total phase								
Bodyweight (g/bird)	L	108	2,079	169	24.6	31.2	0.433	149
ADG (g/bird/d)	L	104	58.0	4.26	1.26	1.08	0.245	701
Feed intake (g/bird/d)	L	108	94.0	5.29	−0.45	0.83	0.586	729
FCR (g/g)	Q	108	1.78	0.04	−0.13	0.03	<0.001	−14.5
					0.07	0.03	<0.001	13.2
Mortality (%)	L	31	5.49	1.65	−20.1	11.3	0.090	190

SE, standard error, AIC, Akaike information criterion; Q, quadratic; L, linear; ADG, average daily gain; FCR, feed conversion ratio.

1) AIC is an estimator of the relative quality of statistical models for a given set of data (smaller is better).

**Table 3:** Regression equation on the influence of flavonoid-rich herbs (in % of diet as fed) on carcass composition of broiler

Response parameter (% of total weight)	Model	n	Parameter estimates			Model estimates		
	
			Intercept	SE Intercept	Slope	SE Slope	p-value	AIC1)
Weight	L	36	68.6	1.12	−2.39	5.33	0.659	164
Breast	L	45	24.8	1.18	5.72	5.42	0.299	195
Liver	L	49	2.42	0.09	0.36	0.14	0.018	43.0
Gizzard	L	40	2.02	0.21	0.17	0.56	0.759	47.5
Abd fat	L	55	1.65	0.10	−0.93	0.67	0.171	20.7

SE, standard error, AIC, Akaike information criterion; L, linear.

1) AIC is an estimator of the relative quality of statistical models for a given set of data (smaller is better).

**Table 4:** Regression equations on the influence of flavonoid-rich herbs (in % of diet as fed) on blood constituents of broilers

Response parameter	Model	n	Parameter estimates				Model estimates	
	
			Intercept	SE Intercept	Slope	SE Slope	p-value	AIC1)
Glucose (mg/dL)	L	12	206	9.81	−28.8	17.9	0.151	93.6
Cholesterol (mg/dL)	L	26	111	13.0	−165	46.6	<0.001	225
Triglyceride (mg/dL)	Q	35	53.7	7.55	−2.64	9.34	0.780	271
					−62.1	25.3	0.022	257
High-density lipoprotein (mg/dL)	Q	30	59.0	7.51	17.0	13.9	0.235	220
					79.0	22.6	0.006	204
Low-density lipoprotein (mg/dL)	Q	30	60.3	13.1	−179	37.8	<0.001	275
					−292	46.0	<0.001	255
MDA (nmol/mL)	L	23	4.67	0.65	−8.20	0.99	<0.001	66.6
Super oxide dismutase (U/mL)	L	24	198	45.8	154	23.6	<0.001	251

SE, standard error; AIC, Akaike information criterion; L, linear; Q, quadratic; MDA, malondialdehyde.

1) AIC is an estimator of the relative quality of statistical models for a given set of data (smaller is better).

**Table 5:** Regression equations on the influence of flavonoid-rich herbs (in % of diet as fed) on small intestinal morphology of broilers

Response parameter	Model	n	Parameter estimates				Model estimates	
	
			Intercept	SE Intercept	Slope	SE Slope	p-value	AIC1)
Duodenum								
Villus height (μm)	L	29	1,488	93.4	1,773	554	0.005	347
Crypt depth (μm)	L	29	228	26.8	97.9	63.5	0.141	251
VH:CD	L	29	7.01	0.80	10.6	4.90	0.045	92.1
Jejunum								
Villus height (μm)	L	35	1,197	116	−12.3	8.56	0.166	428
Crypt depth (μm)	L	35	164	20.1	−2.78	1.15	0.025	299
VH:CD	L	35	8.09	1.20	0.53	0.08	<0.001	141
Ileum								
Villus height (μm)	L	13	677	65.0	211	578	0.725	141
Crypt depth (μm)	L	14	137	7.60	−194	36.7	<0.001	93.6
VH:CD	L	14	5.12	0.79	8.70	4.61	0.096	42.3

SE, standard error; AIC, Akaike information criterion; VH:CD, ratio of villus height and crypt depth; L, linear.

1) AIC is an estimator of the relative quality of statistical models for a given set of data (smaller is better).
